# Leptomeningeal Dissemination in Gall Bladder Carcinoma: Sequelae of Long-Term Survival?

**DOI:** 10.1155/2014/717403

**Published:** 2014-11-05

**Authors:** Shikha Goyal, Bidhu Kalyan Mohanti

**Affiliations:** Department of Radiation Oncology, All India Institute of Medical Sciences, New Delhi 110019, India

## Abstract

Patients with gall bladder malignancies usually present at an advanced stage with less than 20% cases being resectable at presentation and over a half harbouring distant metastases to liver or paraaortic nodes. Long-term cure is uncommon and so is the presence of central nervous system metastases. We present the case of a middle-aged woman with adenocarcinoma gall bladder, treated with postoperative locoregional irradiation following simple cholecystectomy, who developed headache, backache, vision loss, and multiple joint pains six years following adjuvant therapy. A diagnosis of leptomeningeal carcinomatous meningitis was established with cerebrospinal fluid cytology positivity for carcinoma. She deteriorated on palliative cranial irradiation and was managed with best supportive care.

## 1. Introduction

Gall bladder adenocarcinoma is an aggressive disease, with the best surgical series reporting a survival of 63% following an extended cholecystectomy in T3 disease [1]. Survival is dismal in the absence of completion surgery following inadvertent detection on laparoscopic cholecystectomy. Five-year survival in most large series is under 5% and median survival less than six months. Dissemination is early, with over 50%  of cases harbouring metastatic disease at presentation, commonest site being the liver [2].

We present a case of a lady with carcinoma gall bladder treated with laparoscopic cholecystectomy and adjuvant radiotherapy, who developed neoplastic meningitis 6 years following initial therapy.

## 2. Case Summary

A 54-year-old lady presented to our department with a history of low backache for three months, painless progressive loss of vision in both eyes for 25 days, multiple joint pains, and headache for 20 days. There was no history of seizures, altered sensorium, motor, or sensory deficit apart from the aforementioned vision loss. Six years earlier, she had been diagnosed to have well differentiated adenocarcinoma gall bladder T3NxM0 incidentally diagnosed following cholecystectomy, for which she denied completion cholecystectomy and received adjuvant radiotherapy by 3-dimensional conformal radiotherapy. Planning target volume consisted of gall bladder fossa with 2 cm margin and portal, celiac, superior mesenteric, and paraaortic nodal regions. A dose of 45 Gray in 25 fractions was delivered on a Linear accelerator with 6 MV photons using three fields (two wedged lateral opposed portals and one anterior portal conforming to the delineated volumes on CT scan). She had been on regular followup without any evidence of recurrence over the past six years. Her vital signs were unremarkable, apart from mild pallor.  Neurological examination revealed bilateral papilledema on fundoscopy. There was no memory impairment, cranial nerve palsy, or motor or sensory deficit. Visual acuity was 6/60 in both eyes after pinhole correction. Deep tendon reflexes were present and bilateral plantar reflexes were downgoing. There was no neck rigidity. Complete blood counts and serum biochemistry returned normal. Serum electrophoresis, coagulation profile, and vasculitis workup were unremarkable. Cerebrospinal fluid (CSF) analysis showed lymphocytic pleocytosis with increased protein and normal sugar. Repeat CSF cytology showed leucocyte common antigen (LCA) negative and cytokeratin (CK) positive cells suggesting carcinomatous meningitis. Contrast enhanced computed tomography (CECT) of thorax and abdomen showed no lung metastases and multiple paraaortic lymph nodes. Liver and gall bladder fossa were unremarkable. She was given supportive treatment with steroids, diuretics, and pain control measures. She received palliative holocranial radiotherapy (15 Gray in five fractions delivered once daily using two parallel opposed fields on a cobalt-60 unit), but further treatment was discontinued in view of her deteriorating general condition, progressive symptoms, and unwillingness for anticancer therapy and she was referred for hospice care.

## 3. Discussion

Central nervous system (CNS) metastases from gall bladder cancer are rare and may present in a variety of ways. The commonest presentation is leptomeningeal carcinomatous meningitis, described in 10 cases prior to this report [3–12]. In all of these cases, meningitis was the first manifestation and primary disease in the gall bladder was asymptomatic and diagnosed either during a detailed clinical work up or at autopsy.

Symptoms may be due to CSF flow obstruction manifesting as intracranial hypertension, direct infiltration of nerves that cross subarachnoid space leading to cranial nerve palsies, occlusion of pial blood vessels causing stroke-like symptoms, irritation of underlying brain parenchyma leading to seizures, or encephalopathy due to interference with normal CNS metabolism. Headache, altered mental status, cranial nerve palsies, back or radicular pain, incontinence, lower motor neuron weakness, and sensory abnormalities are common presenting findings. CSF cytology is the key to diagnosis, with a yield of 85% with 3 lumbar punctures. Other CSF findings include elevated pressure, low glucose, high protein, and pleocytosis. Contrast enhanced magnetic resonance imaging (MRI) is the most sensitive imaging modality, with findings of meningeal enhancement, hydrocephalus, and spinal subarachnoid masses. Treatment is essentially palliative, with radiotherapy, intrathecal chemotherapy with methotrexate, thiotepa, or cytarabine, or systemic therapy for extracranial disease, depending upon performance status, degree of neurologic deficits, and extent of systemic disease [13].

Other reported CNS presentations include the following.

- First is a left cerebellopontine angle tumor with osteolytic changes in left petrous apex mimicking a tentorial meningioma [14].
- Second is a left frontal hyperdense lesion manifesting with stroke-like features (sudden onset aphasia, altered sensorium, right hemiparesis, hypertension, right facial nerve palsy, and exaggerated deep tendon reflexes). Excision of tumor and evacuation of an organized clot resulted in complete neurologic recovery. Histology showed adenocarcinoma with neovascularisation and further imaging workup showed porcelain gall bladder, with liver metastases, and the patient was kept on best supportive care [15].
- Third is a left frontal cystic lesion with mass effect, presenting with seizures, and right hemiparesis, speech, and memory disturbances, five months following radical surgery for a gall bladder carcinoma. The patient was treated with steroids and tumor excision followed by systemic chemotherapy with cisplatin 30 mg/m^2^ weekly for five weeks, repeated every 3-4 months for one year. She was alive and disease-free at four years [16].

Prognosis is universally poor, with a fulminant course. Only one long-term survival has been reported.

The present case is unique in the fact that the patient had a long disease-free interval of six years despite incomplete surgery, with delayed CNS metastases to the leptomeninges. Presentation with blindness (possibly due to optic atrophy following papilledema) and joint pains in the absence of other cranial nerve palsies is relatively uncommon and confusing. Even in her case, the regional paraaortic disease at recurrence was silent with CNS manifestations dominating the picture.

A high index of suspicion needs to be maintained for all patients with prior malignancy presenting with a myriad of neurologic and nonspecific clinical features to establish a diagnosis of neoplastic CNS spread including carcinomatous meningitis. Individualized therapy may help achieve long-term survival in occasional patients with good performance status, limited CNS involvement and controlled systemic disease, and palliation in others.
